# An approach to identifying young children with developmental disabilities via primary care records

**DOI:** 10.12688/wellcomeopenres.17051.1

**Published:** 2021-07-26

**Authors:** Sarah C. Masefield, Stephanie L. Prady, Kate E. Pickett

**Affiliations:** 1 Health Sciences, University of York, York, Yorkshire, YO10 5DD, UK

**Keywords:** Child disability, developmental disabilities, developmental delay, preschool, electronic records

## Abstract

**Background:** Preschool aged children with developmental disabilities frequently receive a diagnosis of an indicator of disability, such as developmental delay, some time before receiving a definitive diagnosis at school age, such as autism spectrum disorder. The absence of a definitive diagnosis potentially underestimates the need for support by families with young disabled children. Our aim was to develop a two-part strategy to identify children with probable and potential developmental disabilities before the age of five in primary care records for a UK birth cohort, considering how the identification of only probable or potential developmental disability might also influence prevalence estimates.

**Methods:** As part of a study of the effects of caring for young children with developmental disabilities on mothers’ health and healthcare use, we developed a two-part strategy to identify: 1) children with conditions associated with significant disability and which can be diagnosed during the preschool period; and 2) children with diagnoses which could indicate potential disability, such as motor development disorder and developmental delay. The strategy, using Read codes, searched the electronic records of children in the Born in Bradford cohort with linked maternal and child sociodemographic information. The results were compared with national and Bradford prevalence estimates, where available.

**Results:** We identified 83 children with disability conditions and 394 with potential disability (44 children had both a disability condition and an indicator of potential disability). When combined, they produced a developmental disability prevalence of 490 per 10,000 which is above the UK estimate for developmental disabilities in children under five (468 per 10,000) and within the 419-505 per 10,000 prevalence estimated for Bradford (for children aged 0-18).

**Conclusions:** When only conditions diagnosed as developmental disabilities are used for case ascertainment, most of the young children with developmental disabilities likely to be diagnosed at later ages will be missed.

## Introduction

Developmental disabilities are long term physiological impairments that significantly affect a child’s ability to perform activities of daily living, such as independent feeding, mobility, and communication (Unicef and World Health Organization 2012). Globally in 2016, 840 per 10,000 of children under the age of five were estimated to have developmental disabilities ^1^. However, the accurate prevalence estimation of this group of disabilities is influenced by taxonomic and diagnostic decisions and norms in clinical practice and academia and by how conditions recognised as developmental disabilities, e.g. Down syndrome and autism spectrum disorders (ASD), are recorded in healthcare systems. The reliable and accurate estimation of the prevalence and social context of both disabilities and diagnostic practices is necessary for understanding the extent of the burden of disability on individuals and their families for the provision of appropriate health, social care and other supportive services. Awareness of differences in how developmental disabilities are classified and prevalence estimates derived via healthcare systems also provides valuable information for making inter and intra country comparisons; and thus, identifying differences in need. The identification of young children with developmental disabilities can enable earlier support for these children and their families, as is recommended ^2^.

There may be a great deal of inter- or even intra-country variation in prevalence estimates due to different age ranges and conditions being included in the classification of developmental disabilities. For example, the United Kingdom (UK) prevalence of developmental disabilities for children under the age of five years is estimated at 468 per 10,000 ^1^. It includes vision and hearing loss, epilepsy, and attention deficit hyperactivity disorder (ADHD) but excludes motor development disorders, except for cerebral palsy when learning disability is indicated. In the United States (US), the prevalence estimate for 3–17 year olds (an estimate for 0–5 year olds was unavailable) is up to 1,500 per 10,000 ^3^. In addition to the difference in the age range, the US estimate contains a greater range of conditions than the UK estimate, the US estimate includes a greater range of conditions: attention deficit hyperactivity disorder; intellectual disability; cerebral palsy; ASD; seizures; stuttering or stammering; moderate to profound hearing loss; blindness; learning disorders; and/or other developmental delays. Disaggregation of data by age and the conditions identified as developmental disabilities is helpful but not always presented, especially in small studies where participant identification must be avoided. 

For research, electronic health records are an important source of data and clinical codes for diagnoses recorded in primary care records have been used to produce prevalence estimates for people in the UK with learning disabilities and for people who are likely to be disabled ^4, 5^. In the UK, diagnoses of developmental disabilities are usually made by a secondary care specialist (e.g. a Child Development Centre), communicated to the child’s primary care provider via a consultant letter and recorded in the child’s primary care record ^6^. Disability describes how impairment affects function, but electronic health records are based on a system of clinical codes designed to classify disease and conditions, not function (World Health Organization, 2018). The extent of the impact of a condition on function can vary considerably from no impairment to profound. The degree of disability is not usually recorded alongside the diagnosis, unless specified as part of the clinical code e.g. profound learning disability ^4^. Likewise, a child receiving a diagnosis of developmental delay could have a mild, profound or potentially transient disability, but this is not reflected in the clinical codes.

There are two approaches to identifying disability cases from health records: 1) identify those with conditions classified as developmental disabilities (hereafter referred to as disability conditions); or 2) identify those with indicators of potential disability. The first approach will inaccurately identify some, but presumably few, children who do not have disability (false positive) but will miss many children who might (false negative). The second approach will have a higher false positive rate and a lower false negative rate. Allgar *et al.* ^5^ provide an example of the first approach to case ascertainment as they sought to identify only people with a very high likelihood of learning disability, therefore arriving at a conservative estimate of the prevalence of people with learning disability. Lingam *et al.* produced a prevalence estimate for people who potentially have disability, which will have included an unknown number of people without disability and is an example of the second approach to case ascertainment. 

The preschool period (child age 0–5 years) is when parents usually start to notice developmental differences between their child and other children ^7, 8^. It is during this period that they often seek and receive either a diagnosis for a disability condition, such as ASD, or for developmental delay or a developmental disorder, which are indicators of potential disability but are not definitive ^9^. For example, ASD and cerebral palsy can be diagnosed at age 3 years ^9, 10^. However, in practice, it is common for clinicians to wait until children are school age (above five years) to diagnose the disability condition ^11– 14^. For cerebral palsy and learning disability this may be because the diagnostic tests cannot be used accurately before the child is school aged ^15^. For example, learning disability is underdiagnosed in preschool children because an IQ test, the standard assessment used to distinguish mild, moderate or profound learning disability, is not appropriate for use ^16^. Instead, it is standard practice for children aged 0–5 years with developmental disabilities to first receive diagnoses that indicate potential rather than definitive disability, such as developmental delay or disorders relating to specific characteristics (e.g. delayed speech or social interaction) ^17^. The only notable exceptions are a few congenital anomalies, such as Down or Edwards’ syndromes, for which all pregnant women are offered routine pre-natal screening ^18^.

To add further uncertainty, whether and which diagnosis is received during the preschool years is not a reliable indicator of disability severity. For example, a child under five can receive the same diagnosis of developmental delay for either a profound learning disability or if they simply fail to meet their developmental milestones but go on to catch up over time ^19^.

There are relationships between sociodemographic factors and the diagnosis of disability conditions and indicators of potential disability which will affect prevalence estimates, perhaps particularly during the preschool period. For example, low socioeconomic status is associated with an increased risk of developmental delay ^20^. There is a greater risk of Down syndrome in children of older mothers (who also often have high education and socioeconomic status) ^21^; and high maternal education is associated with higher rates of ASD diagnosis ^11^. Pakistani ethnicity is associated with a higher prevalence of congenital anomaly ^22^. Children of ethnic minority mothers are less likely to receive a diagnosis of ASD by age eight years than children of white British mothers (but the true prevalence is not expected to differ between these ethnic groups) ^11^. As sociodemographic contexts vary by place, so too might the accuracy of prevalence estimates and risk of false negative and positive misclassification in the measurement of developmental disability via primary care records.

Some of this variance has known biological explanations, while some may be due to inequalities in accessing healthcare and recording diagnoses. For example, ethnic minority mothers without English language fluency may find it harder to persist in seeking a specific disability diagnosis (e.g. ASD) than, in particular, white British mothers with high education. These factors may influence the extent of the false negative/positive error and thus bias any estimates of the prevalence of developmental disability. For example, children of ethnic minority and low socioeconomic status mothers may be both more likely to receive a diagnosis of an indicator of potential disability rather than a disability condition during the preschool period and less likely to receive any diagnosis.

To our knowledge, no previous research has looked at how many young children receive diagnoses of disability conditions versus indicators of potential disability and the relationship of these to sociodemographic factors. No existing strategies to identify people with disabilities via primary care data are appropriate. Allgar *et al.*’s list of clinical codes would not identify young children with developmental disabilities as even the children with severe learning disability would not yet have received a definitive condition diagnosis and codes for indicators of potential learning disability (e.g. developmental delay) were not included. Lingam *et al.*’s list extends beyond the scope of developmental disabilities. As such one strategy is too narrow and the other not narrow enough to estimate the prevalence of developmental disabilities during the preschool period.

Our aim was to develop a two-part strategy that identified children with probable and potential developmental disabilities diagnosed before the age of five years in primary care data for a UK birth cohort, considering how the identification of only probable or potential developmental disability might influence prevalence estimates.

This study was conducted as part of a PhD research project exploring the health and healthcare use of mothers of young children with developmental disabilities using primary care data linked with sociodemographic data from the Born in Bradford (BiB) cohort study ^23^. As such, much of the research presented here is also available in the lead author’s thesis published in the White Rose eThesis Online repository. 

## Methods

Women were recruited to the BiB cohort between March 2007 and December 2010. The cohort comprises of 12,453 mothers, 13,776 pregnancies and 3,448 fathers, and has been described elsewhere ^24^. We used data from the BiB baseline questionnaire completed when women were recruited to the study linked with primary care records for mother-child dyads for the period 2007–2015.

The BiB study received ethical approval for data collection from the Bradford Research Ethics Committee (Ref 07/H1302/112). Our study received ethical scrutiny as part of our BiB data application, and we complied with all standards and policies of the University of York’s Data Management Policy (University of York Information Services, 2018). As our study was a secondary analysis of an existing data set, additional ethical approval was not needed. 

### Strategy development

We developed a two-part strategy to identify children aged 0–5 years via electronic primary health records: 1) with a disability condition; and 2) with an indicator of potential disability. The strategy was developed following consultation with paediatric clinical researchers at the University of York (Dr Bob Phillips and Professor Lorna Fraser) and paediatric clinicians in the Bradford Child Development Centre (Dr Stella Yeung and a Lead Nurse in the Child Development Service).

The first part aimed to identify children with the most common (prevalence of at least one in 10,000 children aged 0–18 years) conditions that cause significant long term variation in the child’s capacity to achieve the expected developmental (functional performance) milestones for their age (World Health Organization, 2012) and can be diagnosed below the age of five years. We used the developmental disabilities most frequently associated with paediatric disability complexity by Horridge *et al.* ^25^: ASD, cerebral palsy, chromosomal syndromes and intellectual disability ( Table 1). The specific chromosomal syndromes of Down syndrome and Fragile X syndrome were specified as these are the two most common chromosomal syndromes which typically cause disability ^5^. Learning disability is one of the few conditions classified by severity (from mild to profound) in the clinical coding hierarchy and was restricted to moderate-profound severity.

**Table 1.** United Kingdom (UK) prevalence estimates and disability characteristics for the disability conditions.

Disability condition	Prevalence estimate ^1^	Disability-related factors (typical and common)
Moderate, severe and profound learning disability	• 350 per 10,000 (aged 5–18 years) (300 moderate, 37 severe, 13 profound) (Public Health England, 2018; Hatton, 2016)	• Learning disability (the inability to understand and perform daily activities) • Behavioural problems (common)
ASD	• 38 per 10,000 boys aged 8 years (3 years for girls) (Taylor, 2013) • 103 per 10,000 children aged 5–8 years in Bradford (Kelly, 2017b)	• Delayed speech and social interaction problems (typical) • Learning disability (if severe ASD) and behavioural problems (common)
Cerebral palsy	• 20 per 10,000 children aged 0–5 years (Cans, 2008) • Up to 41 per 10,000 children aged 0–5 years in Bradford (Sinha, 1997)	• Motor impairment (typical) • Learning disability and behavioural problems (common)
Down syndrome	• 9 per 10,000 children aged 0–5 years (Alexander, 2016)	• Learning disability (typical) • ASD and behavioural problems (common)
Fragile X syndrome	• 2 per 10,000 aged 0–10 years (3 years for boys, 1 year for girls) identified via pre-natal screening (Song, 2003)	• Learning disability (typical) • ASD and behavioural problems (common)
Combined prevalence for the conditions	• 419 per 10,000 • 505 per 10,000 for Bradford	

ASD; Autism Spectrum Disorders ^1^ UK prevalence estimates for children aged 0–5 years were not available for every condition (estimates provided as integers). The youngest age range possible is given and estimates for Bradford provided, where available. Where there are differences in prevalence by sex, disaggregated estimates are provided.

The second part of the strategy reflected the practice of deferred disability diagnosis identified by the Bradford-based clinicians, that whilst the disability conditions can be diagnosed in children under five, it is common practice for children in Bradford (and elsewhere) to receive these diagnoses later (age 5 years and above). Therefore, we also aimed to identify children with indicators of potential disability classified as: developmental delay; generalised developmental disorders; disorders relating to specific developmental characteristics; mild or unknown severity learning disability; and generic disability (e.g. on learning disability register and disability not otherwise specified).

Each part consisted of four code lists: four for the disability conditions (n=148 Read codes) ( Table 2A) and four for the indicators of potential disability (n=103 Read codes) ( Table 2B).

**Table 2.** Read code lists for case ascertainment: probable disability (2A); potential disability (2B).

Table 2A. Disability conditions code list											
Cerebral palsy											
XE2Q8	XE15M	X00En	Xab3R	XaYgp	XaYfK	X00Eo	XE2se	XM1Pw	XE2Q9	F2300	F230z
X00Ep	XM1Px	F230.	X00Eq	F231.	F234.	XE15V	X00Er	X00Es	XM1Pv	X00Eu	XaadE
XE2Q7	X00Ew	Xa0lM	F23y0	Xa0lI	X00Ex	F23y1	X00Ey	X00Ez	XaNWb	X00F1	X00F2
X00F3	F23y.	F23yz	F23z.	X00Em	Fyu90	XM1Pu	XaBE2	F1371	F23..	XE181	F23y0
Xa0lM	F2B2.	Xab3R	.F32Z	F23y.	F23yz	F23z.	F2B..	F2By.	F2Bz.	Fyu90	X00Em
F23y6	XaadE	XM1Pu	F23y3	X00Eu	F2301	F23y2	X00En	XE2Q9	XM1Pv	XaaVG	XaaWF
XaaVJ	XaaWE	XaaVK	XaaWD	XaaVI							
Down syndrome											
.N721	XE1MZ	PJ00.	PJ01.	PJ02.	X78El	PJ0z.	X78Ek	XE1MZ			
Fragile X syndrome											
X78FB	PJyy2	X78FC	X78FD								
Autism Spectrum Disorders											
X00TM	XaesO	XE2v2	E1400	E1401	E140z	X00TN	X005S	E141.	E1410	E1411	E141z
X00TP	Ub1Ts	Eu844	Eu84y	Eu84z	XE1aA	E140.	Eu840	Eu841	Eu84.	Eu84y	Eu845
.E2Z3	Eu844	XE1aA	Eu84z	Ub1Tr	Ub1Tw						
Mod-severe learning disability											
E310.	Eu710	Eu711	Eu71y	Eu71z	E311.	Eu720	Eu721	Eu72y	Eu72z	E312.	Eu730
Eu731	Eu73y	Eu73z	Xa3HI	Eu7y1	Eu7z1	XaREu	Xabk1	Xa00k	Eu73.	Eu71.	.E512
Xa01E	.E513	Eu72.	Xa00l								
Table 2B. Indicators of potential disability code list											
Developmental delay											
X76B7	XaX18	Ua14s	Xa40J	XaXCG	XaBBv	E2F..	E2Fy.	XaIsc	XaO45	XaO46	XaO47
Ub1US	XacSD	Ub1UM	Ub1UO	Ub1UQ	E2E1.	Xa09f	Ub1U6	Ub1U2	R0340		
Generalised developmental disorders											
X00TQ	XE1Z4	XM1MS	X00TI	Eu8..	XE1Z3	XE1a4	XE1a3	Ub1UL	E2F3z	X00TK	XE1a6
XE1a7	XE2bB	XE1Z5	Ub1Tf	E2F5.	E2Fz.	Eu83.	Eu8z.	XE1aB	Ub1S4	X00F0	XM0zA
XE1gX	XM1AJ	Ub1UG	XacL0	XacKx	Ub1UR	Ub1UT	Ub1UU	Ub1UV	Ub1UW	Ub1UX	XE1a5
Ub1U0											
Generalised disabilities											
E3...	XE2a3	Eu700	Eu701	Eu70y	Eu70z	Xa0ER	Xa3HI	E31..	E31z.	Eu7y0	Eu7y1
Eu7yy	Eu7yz	E3z..	Eu7y.	Eu7z0	Eu7z1	Eu7zy	Eu7zz	XE1a2	XabmM	XacF5	X00TL
XaaiS	XacF6	XaREt	Eu813	E2F2.	Eu81.	Eu81z	XE1a9	13ZK.			
Generic disability											
13VC5	13VC1	13VC2	13VC3	13VCZ	XaKYb	XaDyv	.6664	6665.	9EB4.	6972.	

The lists used the hierarchical clinical code language Clinical Terms Version 3 (commonly known as Read codes) as all primary care practices in Bradford use the SystmOne electronic record system ^26^. They were developed using the National Health Service (NHS) Clinical Terminology Browser Clinical Terms Version 3 - Clinical 2017-10-01 Drugs 2016-04-01 (also known as a Read code browser). Only Read codes which positively identified the condition or indicator were included in the lists. They were identified by searching for the condition key term (e.g. Down syndrome), then using the step-up/step-down functions to identify all relevant Read codes in the ‘Clinical findings: Disorders’ hierarchy of the classification system.

Drug, treatment and referral Read codes were not included. These codes indicate potential disability complexity, including chronic illness, but do not on their own provide enough information to deduce disability. Codes for assessment were included only when the outcome was a definitive diagnosis of one of the disability conditions. For example, the paediatric consultants recommended including the Gross Motor Function Classification System (GMFCS) for cerebral palsy. The codes for the Surveillance of Cerebral Palsy Europe (SCPE) classification system for cerebral palsy were excluded as the assessment is not used in preschool children and the GMFCS is the preferred assessment tool in Bradford. 

The primary care records of all children in the BiB cohort were searched to identify every child who had one or more of the codes recorded in their primary care record during the period of birth to their fifth birthday. The clinical codes and date of entry for every code were extracted and the age of the child when each code was recorded was calculated to explore differences between when disability condition and indicator codes were received. To protect the anonymity of the study participants, these calculations used the month and year of the child’s birth, using the first date of the month for the calculation. Only child per mother was included, with further exclusions for children who were withdrawn from the BiB study or died, did not have linked primary care data or a maternal BiB baseline questionnaire (n=2,469) ^23^. For every child, we also extracted data on the child’s sex, mother’s age at the child’s birth, ethnicity, measures of socioeconomic status, such as education. Where there were fewer than five children with any of the disability conditions, the children were excluded from the study to protect their anonymity.

### Prevalence

Data analysis was performed using Stata 15 ^27^. Descriptive statistics were used to describe and compare the prevalence of developmental disabilities and sociodemographic differences between the two parts of the case ascertainment strategy.

There is not a gold standard strategy to identify developmental disability in primary care records against which to validate our strategy. As we do not have accurate estimates for the true prevalence in our dataset or the preschool age group in the UK, we compare the prevalence estimates in this dataset to the available Bradford and UK estimates for specific disability conditions presented in Table 1 and an estimate of developmental delay for three year olds in the Millennium Cohort ^20^.

Based on the estimates in Table 1 for children under five, where available, the UK prevalence is 419 per 10,000. However, prevalence estimates also vary by country and region, with a higher prevalence of childhood disability found in Bradford (Wright, 2013). A higher prevalence of ASD and cerebral palsy has been found for Bradford compared with other UK estimates; and a higher prevalence of chromosomal syndromes (per 10,000): BiB cohort 25 versus UK prevalence 15 (Bishop, 2017). This estimate includes Down and Fragile X syndromes but is not disaggregated by condition, so the elevated prevalence of these conditions in the BiB cohort is unknown. Given the known higher prevalence of some conditions in Bradford ( Table 1), the prevalence estimate for this geographical area is (at least) 505 per 10,000.

Most prevalence estimates, including all those presented in Table 1, are dependent on the children receiving diagnoses for the disability conditions before the age of five. Lingam *et al.* (2012) found a potential disability prevalence of 130 per 10,000 in children aged 0–4, increasing to 500 per 10,000 for the 5–9 age group. This suggests that we may find the prevalence of both disability conditions and indicators of potential disability in children aged 0–5 identified via primary care records to be substantially lower than both the UK and Bradford prevalence estimates (presented in Table 1).

The prevalence of developmental delay in high income countries is estimated at 300 per 10,000 of children (Leonard, 2002), and was 320 per 10,000 for children aged three years in the UK Millennium Cohort (Emerson, 2008). The prevalence of global developmental delay, where children have a delay in more than one area of development e.g. motor and speech, is 100–300 per 10,000 (Mithyantha, 2017). The second part of our case ascertainment strategy was expected to identify at least 384 children in the BiB cohort (3% of 12,000), and at least 120 with more than one indicator of potential disability (as a measure for global delay). Given the clinical norm of initially diagnosing developmental delay or a generalised disorder, it was likely that a high proportion of the children identified by the primary strategy would also have indicators of potential disability. The number of codes and the code description found in the records of the children identified as having disability conditions were compared with those of the children with indicators of potential disability only.

We expected sociodemographic differences between the children and the parents identified via the two parts of the strategy: 1) mothers of children in the disability condition group were expected to be older on average (and have higher socioeconomic status) than the potential disability group due, in part, to the relationships between higher maternal age and the increased prevalence of Down syndrome and diagnosed ASD; 2) the age of the children when they received their condition or indicator diagnosis was expected to be lower in the condition group because Down syndrome and Fragile X syndrome are usually identified during pre-natal screening (NHS Choices, 2017) and greater disability severity (including more visible disability) was expected to be associated with earlier diagnosis; and 3) the disability condition group was expected to have a higher proportion of boys than the potential disability group due to the higher prevalence of ASD and Fragile X syndrome in boys (Taylor, 2013; Song, 2003). We performed tests of between group difference for the sociodemographic factors in which we expected the two groups to vary.

## Results

**Figure 1.**
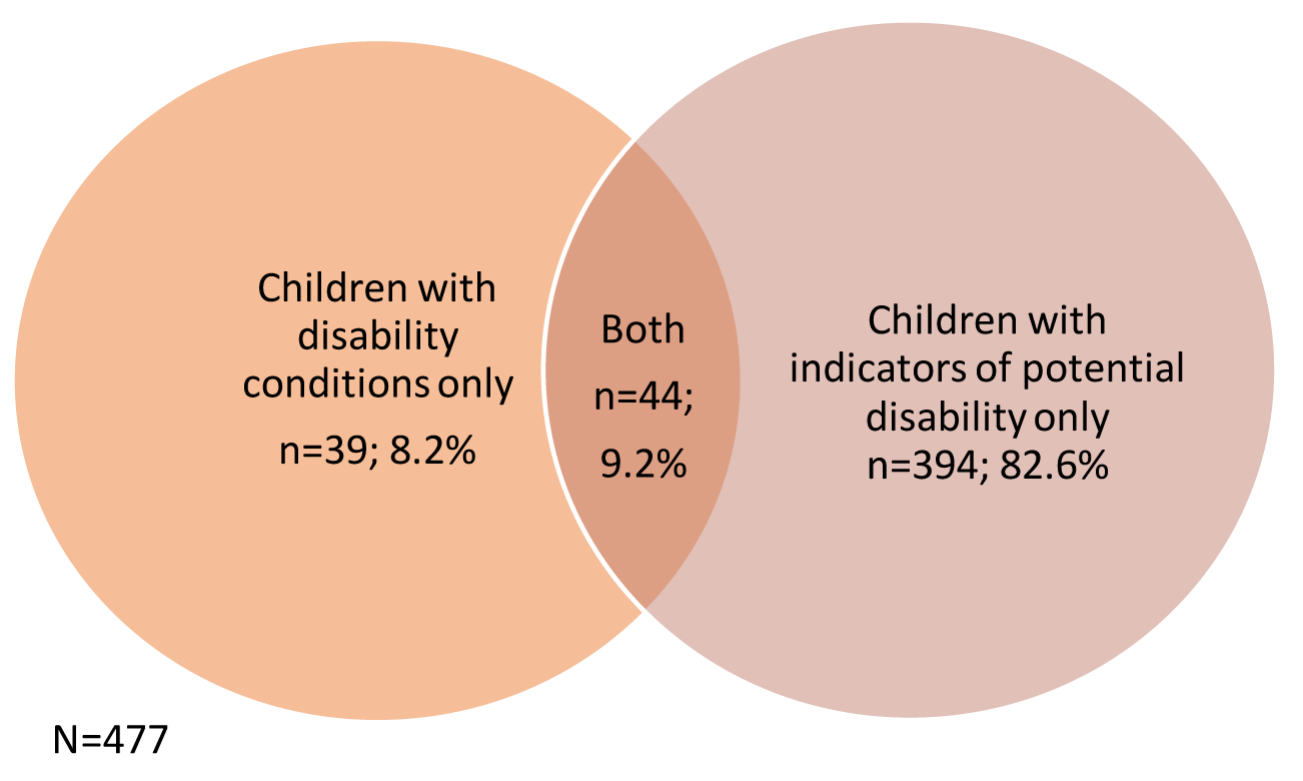
Number of children identified as having probable or potential disability (N=477).

Of the 9,727 children included in the linked study, 477 (4.9%) had either a disability condition (probable disability) or an indicator of potential disability or both ( Figure 1).

The two strategies combined produced a developmental disability prevalence of 490 per 10,000. This is within the 419–505 per 10,000 prevalence estimated for Bradford and above the UK estimate for developmental disabilities (468 per 10,000) ( Table 3).

**Table 3.** Comparison of the United Kingdom (UK) and Born in Bradford (BiB) prevalence of potential and probably disability (per 10,000).

Condition	UK ^1^	Bradford	Born in Bradford ^3^
Disability conditions	419 ^2^	505	85
Moderate-profound learning disability	350 (aged 5–18 years) (Public Health England, 2018)	-	0
Autism Spectrum Disorders (ASD)	38 (aged 8 years) (Taylor, 2013)	103 (aged 5–8 years) (Kelly, 2017b)	48
Cerebral palsy	20 (Cans, 2008)	41 (Sinha, 1997)	12
Down syndrome	9 (Alexander, 2016)	-	25
Fragile X syndrome	2 (Song, 2003)	-	0
Indicators of potential disability (a proxy for developmental delay)	320 (aged 3 years) (Emerson, 2008)	-	450 ^4^

^1^ Denominator of 10,000 used for comparison as close to the sample size. The estimate is for children aged 0–5 years unless stated otherwise. For cerebral palsy, the estimate is per 10,000 live births ^2 ^Combined prevalence of the disability conditions. ^3 ^BiB prevalence below 5 for the study sample was rounded down to protect participant anonymity. ^4 ^Calculated from the number of children with Read codes for potential developmental disabilities (n=438).

### Probable disability

Of the 9,727 children, 83 (0.9%) had a Read code for ASD, cerebral palsy or Down syndrome recorded in their primary care record between birth and age five, giving a prevalence of 85 per 10,000. There were no children diagnosed with moderate-profound learning disability. To protect anonymity due to small numbers, the children with a diagnosis of Fragile X syndrome were excluded from the study.

**Figure 2.**
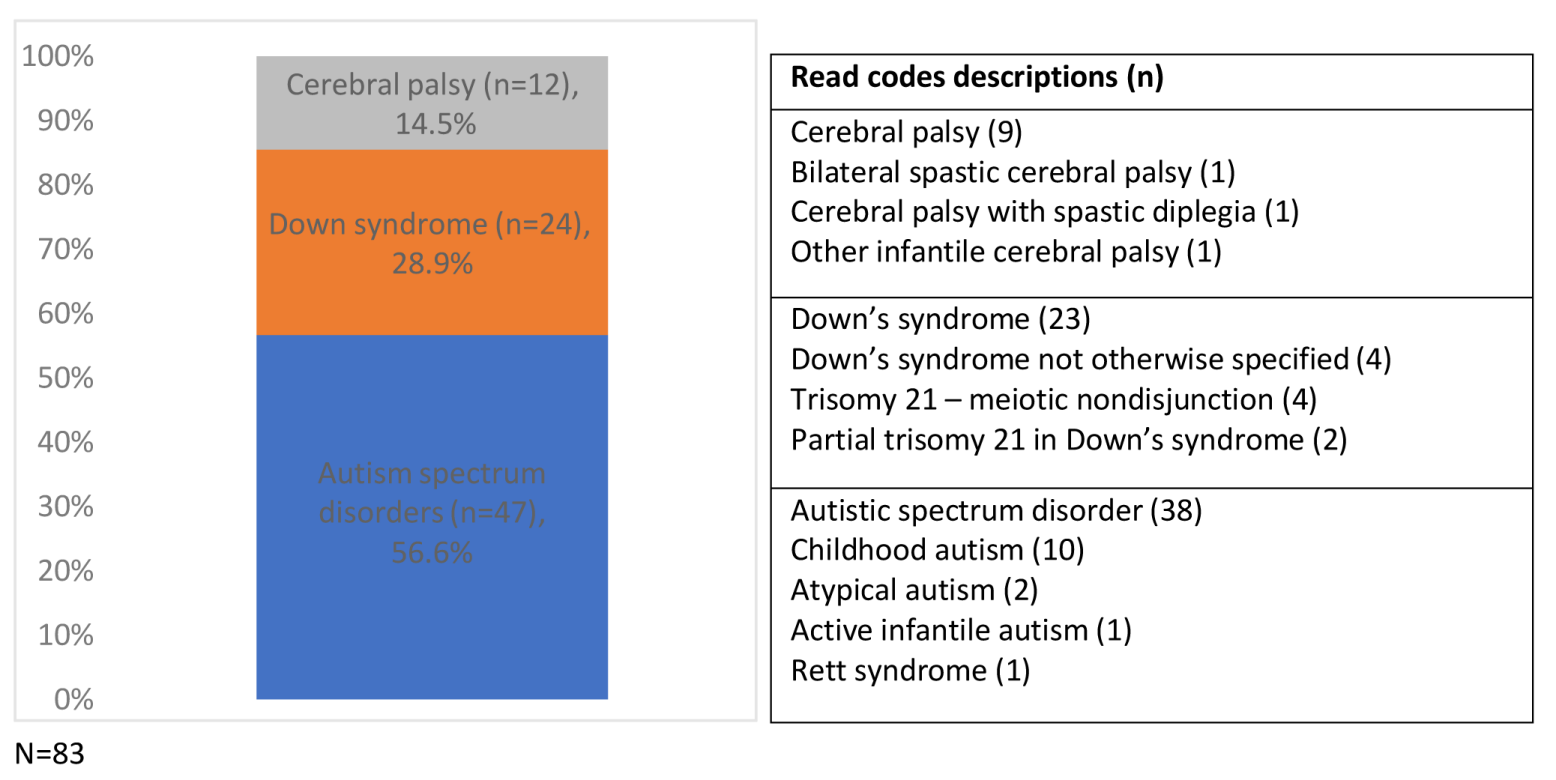
Composition of the probable disability group and frequency of identifying Read codes (N=83). The frequency of each code is not equal to the number of children with each condition as 24 children had more than one code for the same disability condition (the same or different codes) recorded on the same (n=3) or different dates (n=21) during the five year study period.

Of the 148 Read codes searched for, 13 (recorded 97 times) were found in the primary care records ( Figure 2).

**Figure 3.**
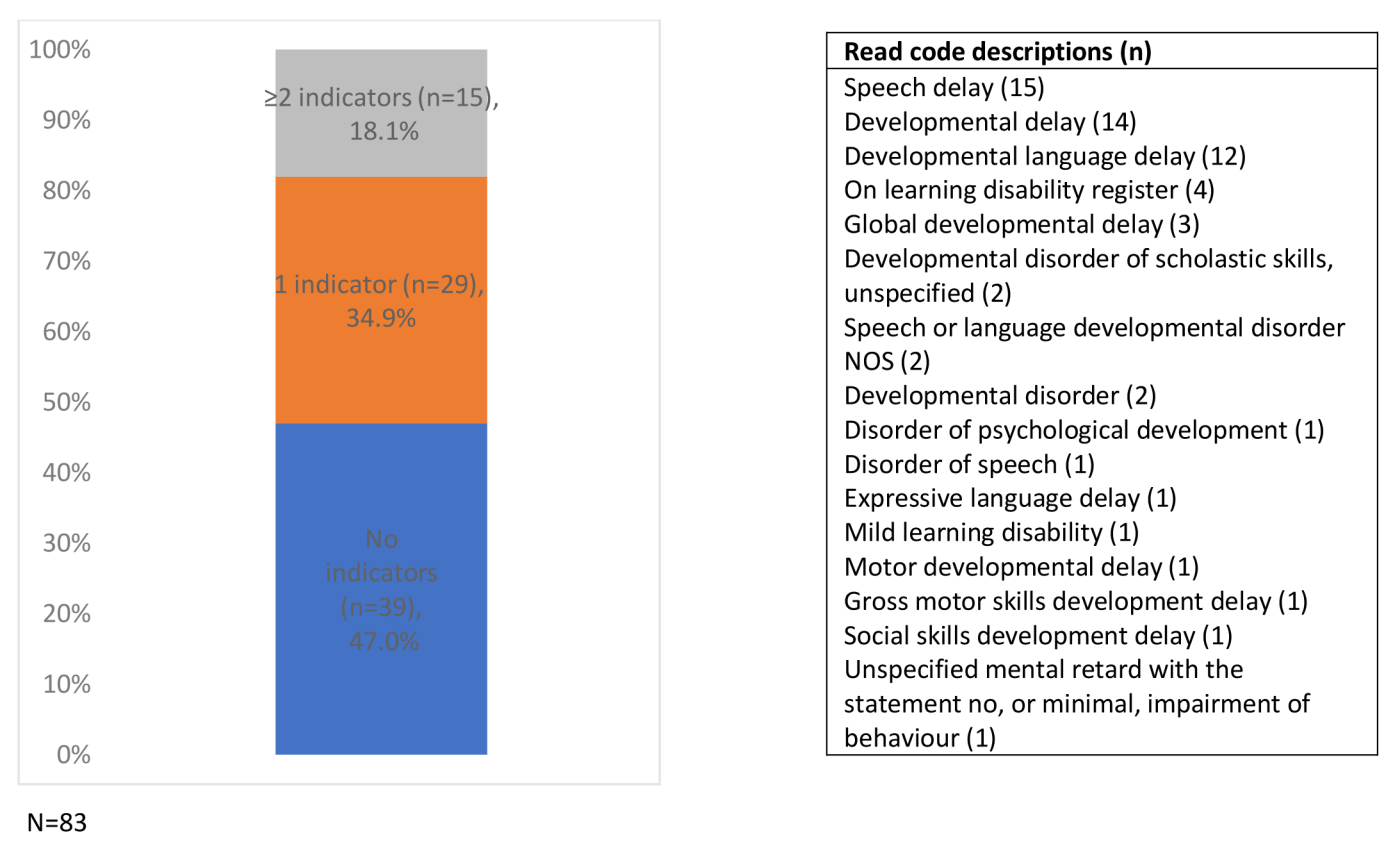
Frequencies of indicators of potential disability in children with probable disability and identifying Read codes (N=83). NOS; not otherwise specified. The same indicator was recorded on more than one occasion during the five-year study period for five children.

No children had more than one of the disability conditions, but 53% (n=44/83) had at least one indicator of potential disability ( Figure 3). Of the 103 Read codes included in the secondary case ascertainment strategy, 16 (recorded 62 times) were found in the children’s primary care records.

As anticipated, the children with Down syndrome received their diagnoses earliest (soon after birth) and the children with ASD received diagnoses latest ( Table 4); and a large proportion of children with ASD and cerebral palsy received a diagnosis of developmental delay prior to receiving a condition diagnosis. There was considerable variability in the age at which children with ASD and cerebral palsy received their first diagnosis (of either a condition or indicator). 

**Table 4.** Diagnostic and sociodemographic characteristics of the mother-child dyads by (probable) disability condition group.

Variable	Cerebral palsy (n=12)	Down syndrome (n=24)	Autism Spectrum Disorders (n=47)	Total (n=83)
Children diagnosed with an indicator before receiving a disability condition diagnosis, n column (%)	6 (50)	0 (0)	17 (36.2)	23.0 (27.7)
Child’s age when a disability condition is diagnosed (in months), mean (s.d.), range	29.6 (19.5), 0–58	0.3 (0.7), 0–3	48.7 (7.6), 32–60	32.0 (23.2), 0–60
Child’s age when first disability condition or indicator is diagnosed (in months), mean (s.d.), range	20.4 (18.3), 0–58	0.3 (0.7), 0–3	39.3 (13.0), 7–60	25.3 (21.0), 0–60
Sex, male, n column (%)	5 (41.7)	12 (50)	37 (78.7)	54 (65.1)
Mother’s ethnicity, n column (%) White British Pakistani Missing	5 (41.7) 7 (58.3) 0	16 (66.7) 8 (33.3) 0	27 (57.4) 20 (42.6) 0	48 (57.8) 35 (42.2) 0
Mother’s highest educational qualification, n column (%) Higher education (beyond age 16) Compulsory education (to age 16) Missing	6 (50.0) 6 (50.0) 0	11 (45.8) 12 (50.0) 1 (4.2)	31 (66.0) 16 (34.0) 0	48 (57.8) 34 (41.0) 1 (1.2)
Mother’s age (in years) at child’s birth, mean (s.d. ^1^), range	24.8 (6.6), 18–41	34.1 (8.1), 18–49	28.2 (5.3), 18–39	29.4 (7.1), 18–49

^1^ s.d.; standard deviation

Compared with the other disability condition groups, the ASD group had a higher proportion of male than female children, mothers who were white British and educated above age 16 ( Table 4). The average maternal age of the Down syndrome group was higher, but there was not a greater proportion of Pakistani (versus white British) or high (versus low) educated mothers compared with the other groups.

### Potential disability

**Figure 4.**
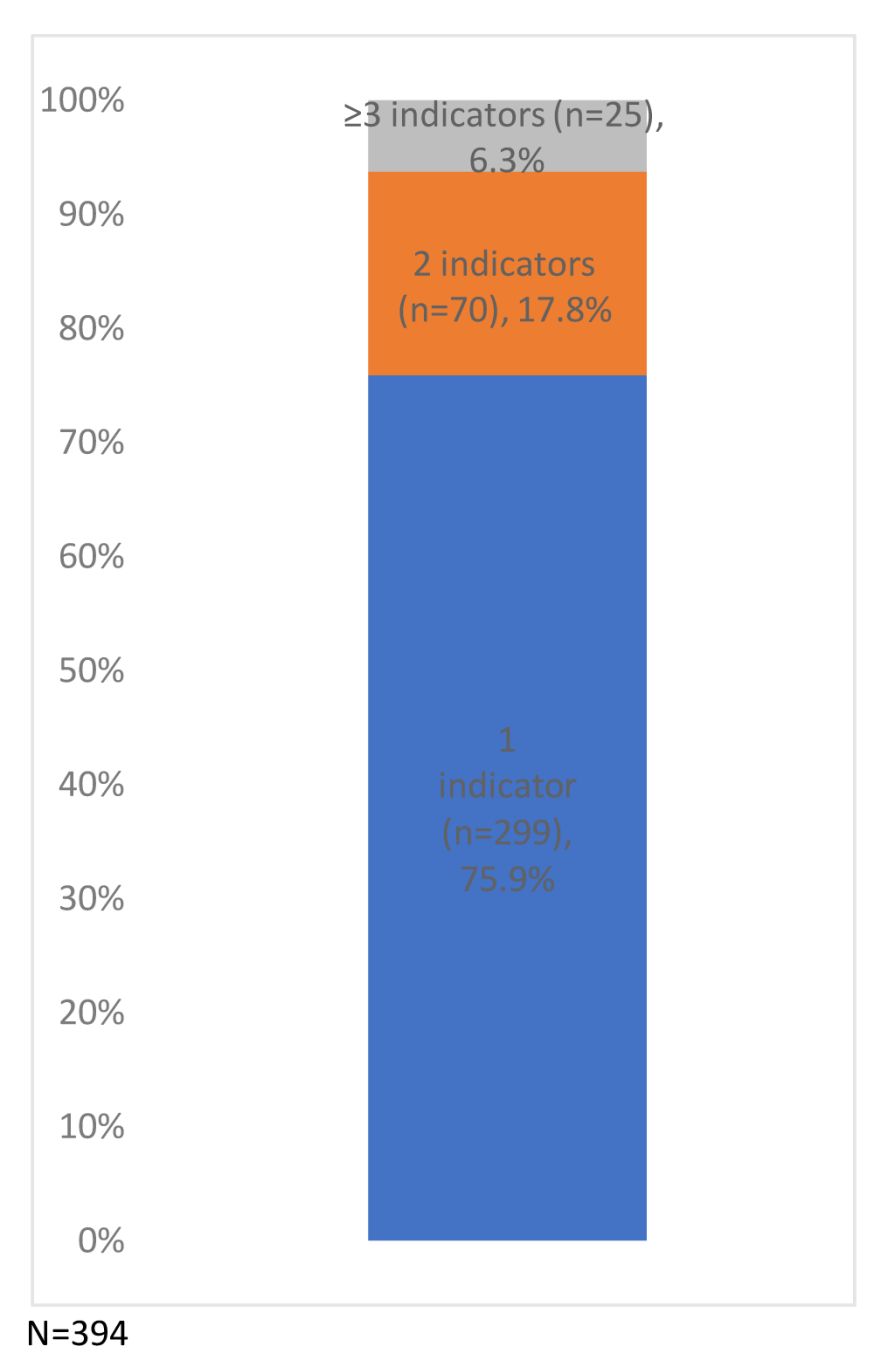
Percentage of children with one or more indicator of potential disability (N=394).

**Figure 5.**
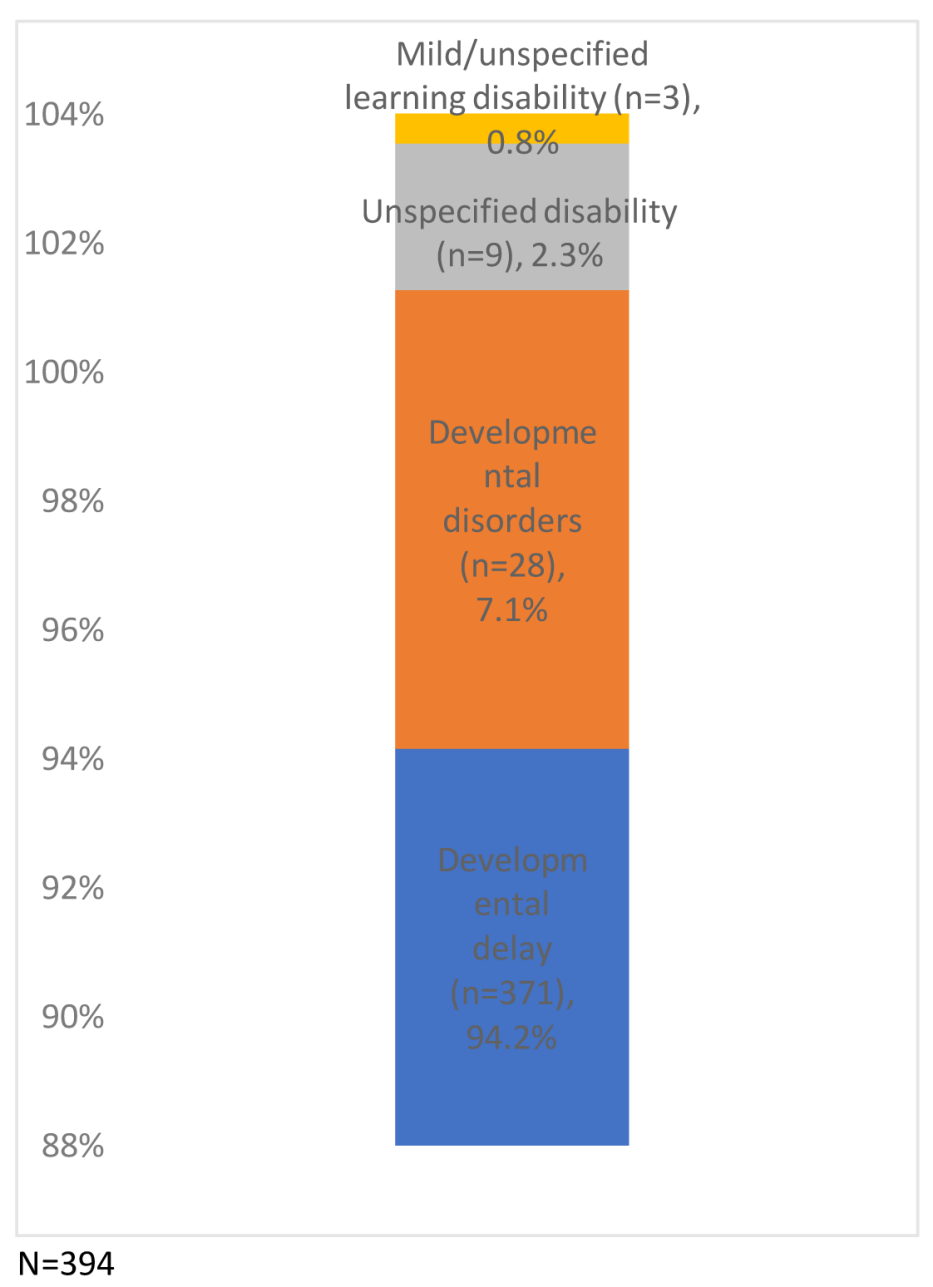
Number of children with a Read code from the potential disability categories (N=394). The categories are not mutually exclusive.

Of the study sample, 4.1% of the children had indicators of potential disability (n=394/9,727), a prevalence of 405 per 10,000 ( Figure 4). Just under a quarter (24.1%) had more than one indicator (from the same or different categories: developmental delay, developmental disorders, mild/unspecified learning disability or other unspecified disability) ( Figure 5).

Of the 103 Read codes in the secondary case ascertainment strategy, 33 (recorded 521 times) are found in the children’s primary care records ( Table 5).

**Table 5.** The frequency of each indicative Read code by potential disability category.

Read code descriptions (n)	
Mild/unspecified learning disability: On learning disability register (2) Mild mental retardation, IQ in range 50–70 (1)	Developmental delay: Speech delay (151) Developmental delay (134) Developmental language delay (101) Global developmental delay (21) Expressive language delay (16) Gross motor skills development delay (15) Motor developmental delay (10) Receptive language delay (5) Development delay NOS (5) Specific delays in development (5) Phonological delay (3) Communication skills development delay (3) Growth delay (3) Other development delays (3) Fine motor skills development delay (2) Social skills development delay (1) Delayed milestone (1) Neurodevelopmental delay (1)
Unspecified disability: DLA 370 Disability living allowance completed (6) Disability NOS ^1^ (1)	
Developmental disorders: Disorder of speech and language development (12) Speech or language developmental disorder NOS (5) Developmental disorder of motor function (3) Developmental disorder (2) Developmental disorder of scholastic skills, unspecified (2) Developmental disorder of speech and language, unspecified (2) Expressive language disorder (1) Developmental disorder NOS (1) Developmental language impairment (1) Developmental language disorder (1) Developmental speech disorder (1)	

^1^ NOS, Not otherwise specified

Clinical codes for general developmental delay or delay in speech and language development occurred most frequently in the children with potential disability ( Table 5) as well as those with disability conditions ( Figure 3).

### Between-group sociodemographic differences

As anticipated, the disability condition group had significantly more highly educated, older mothers and the children received an earlier diagnosis than the potential disability group ( Table 6). Although there is a greater proportion of males in the condition than potential disability group the difference is not significant.

**Table 6.** Sociodemographic characteristics where significant variation was theorised between the potential and probable disability groups.

Variable	Potential disability only (n=394)	Probable disability (n=83)	Tests of difference, test statistic (p-value) ^1^
Child’s sex, n column (%) Female Male Total	114 (28.9) 280 (71.1) 394 (100)	29 (34.9) 54 (65.1) 83 (100)	1.2 (0.28)
Mother’s education, n column (%) Higher education (beyond age 16) Compulsory education (to age 16) Missing Total	182 (46.2) 212 (53.8) 0 394 (100)	48 (57.8) 34 (41.0) 1 (1.2) 83 (100)	**4.1 (0.04)**
Mother’s age (in years) at child’s birth, mean (s.d. ^2^), range	27.4 (5.7), 15-43	29.4 (7.1), 15-44	**-2.1 (0.03)**
Child’s age (in months) at first diagnosis ^3^, mean (s.d.), range	34.8 (14.3), 0-59	24.9 (20.8), 0-59	**3.9 (0.00)**

^1^ Pearson chi ^2^ test was used for categorical variables. The t-test was used for the continuous variables. Two-sided p values were reported. Missing values were excluded from the tests. Statistically significant results are in bold (p<.05). ^2 ^s.d.; standard deviation ^3 ^For the probable disability group, this was a disability condition or indicator depending on which diagnosis was received first.

There were no significant differences for the characteristics in which the groups were not expected to vary ( Table 7).

**Table 7.** Sociodemographic characteristics in which the potential and probable disability groups were not expected to vary.

Variable	Potential disability (n=394)	Probable disability (n=83)
Parity, n column (%) First child ≥2 children Total	358 (90.9) 36 (9.1) 394 (100)	77 (92.8) 6 (7.2) 83 (100)
Cohabitation status, n column (%) Living with partner Not living with partner Total	328 (83.3) 66 (16.8) 394 (100)	72 (86.8) 11 (13.3) 83 (100)
Mother’s ethnicity, n column (%) White British Other Pakistani Missing Total	159 (40.4) 1,462 (15.8) 4,040 (43.7) 19 (0.2) 394 (100)	34 (41.0) 14 (16.9) 35 (42.2) 0 (0.0) 83 (100)
Subjective financial status, n column (%) Living comfortably Doing alright Just about getting by Quite difficult Very difficult Missing Total	82 (20.8) 176 (44.7) 97 (24.6) 23 (5.8) 10 (2.5) 6 (1.5) 394 (100)	25 (30.1) 34 (41.0) 18 (21.7) 4 (4.8) 2 (2.4) 0 83 (100)
IMD ^1^ quintiles, n column (%) 1 (highest SES ^2^) 2 3 4 5 (lowest SES) Missing Total	5 (1.3) 10 (2.5) 33 (8.4) 68 (17.3) 278 (70.6) 0 394 (100)	1 (1.2) 4 (4.8) 11 (13.3) 18 (21.7) 49 (59.0) 0 83 (100)

^1^ IMD; Index of Multiple Deprivation ^2^ SES; Socio-economic status

## Discussion

We developed a two-part strategy to identify children with probable and potential developmental disabilities diagnosed before the age of five in primary care data for a UK birth cohort. Using this strategy, we found that the prevalence of developmental disability in preschool children might be greatly underestimated if only disability conditions are used (85 rather than 419 per 10,000), as is usually the case in research ^1, 28^. The prevalence of the disability conditions was lower than anticipated (except for Down syndrome and ASD). However, when the disability condition strategy that identifies children with diagnosed developmental disability is used together with a strategy that identifies children with potential developmental disability, the resultant prevalence (490 per 10,000) is within the 419-505 per 10,000 prevalence estimated for Bradford and above the UK estimate for developmental disabilities (468 per 10,000).

Many of the children with the disability conditions (excluding Down syndrome) received an initial diagnosis of an indicator of potential disability (36%; n=17 of the ASD group; 50% of the cerebral palsy group). The prevalence of potential disability appeared superficially to be higher than in other samples, such as the 320 per 10,000 prevalence of developmental delay in the UK Millennium Cohort (n=12,689 children aged 3) ^20^. However, that sample consisted of only monolingual English-speaking families as the multilingual families had extremely high rates of developmental delay. The BiB cohort includes multilingual families, and we used a different sampling strategy (clinical codes in electronic health records rather than cross-sectional assessment). Given these differences and the broader age range in our study, it is likely that the prevalence in the cohorts are roughly equivalent.

An additional finding of note was that fewer children in the potential disability group than expected had more than one indicator (n=90 versus the 120 expected) which gives an indication of global development delay (Mithyantha, 2017). This is highly unlikely to mean milder or more transient developmental delay than observed elsewhere, rather it may reflect issues with the identification of global developmental delay or of long intervals between the initial diagnosis of a delay and follow up assessment. It probably also reflects the paediatric clinicians’ anecdotal evidence that when there are signs of developmental disability in a preschool child, an initial diagnosis of developmental delay is given, and a more definitive diagnosis sought after the age of five years.

The practice of deferring giving a definitive (condition) diagnosis until the child is older could explain why there were no or very few children with moderate-severe learning disability or Fragile X syndrome in the cohort. Accordingly, it was highly likely that some of the children in the sample who received indicator diagnoses before the age of five had, as yet, undiagnosed ASD, cerebral palsy and moderate-profound learning disability. It might reasonably be assumed, therefore, that the 83 children in our sample who did receive a disability condition diagnosis before the age of five either had severe disability or a very typical manifestation which made diagnosis straightforward. The possibility of greater disability severity in this group may be supported by the finding that over half (53%, n=44) of the children with disability conditions also had an indicator of potential disability compared with 24% (n=95) of the potential disability group having two or more indicators.

Alternatively, sociodemographic factors may have influenced the diagnosis. In particular, we found, as expected, that a greater number of mothers of children with ASD had higher education than mothers of children with other disability conditions or indicators. This may be due to higher educated mothers being more assertive or persistent in the pursuit of a diagnosis for their child ^11, 29^. An unexpected finding was that there were not more Pakistani than white British children with Down syndrome, despite the prevalence of other congenital anomalies being higher in Pakistani families in Bradford ^12, 22^. The explanation could be that Pakistani mothers in the cohort tended to be younger than the white British mothers. This would reduce the risk of Down syndrome in the babies born to Pakistani mothers given the known association between maternal age and Down syndrome.

### Strengths and limitations

We developed a practical strategy for identifying preschool children with developmental disabilities via primary care records and have identified the practice of deferring the diagnosis of specific developmental disabilities. Without including indicators of potential disability in case ascertainment strategies, young children with developmental disabilities will not be identified, and therefore, would be underrepresented in any prevalence estimates or in research requiring the identification of these children. Only a hybrid strategy which includes Read codes for probable and potential disability could accurately identify the true number of children in the preschool age group with developmental disabilities via primary care records. Whilst our strategy aimed to achieve this, some limitations remained.

The two parts of the strategy were developed to try and balance the risk of including versus excluding an unknown number of children without disabilities. Neither strategy could eliminate the risk of false positives or negatives misclassification entirely, with a greater expected risk of misclassification for the potential disability strategy. However, in practice, this risk was low as it was expected that a disability condition or indicator of potential disability would, largely, only be diagnosed during the preschool period if the characteristics were distinct, which is more likely for moderate and severe than mild impairment. Sensitivity analysis to assess and compare the extent to which the case ascertainment strategies resulted in misclassification error (false positive and false negative) was not performed as this would have required the use of a gold standard comparison strategy. None of the existing strategies were suitable or could be swiftly adapted solely to gauge the extent of the misclassification error. Attempts were made to identify differences in disability severity by measuring the number of diagnoses and age of the child when the mother’s symptoms were detected but no inferences about disability severity could be made.

Further research is required to improve case ascertainment via primary care records for this age group, such as identifying how disability severity can be inferred using only routinely collected data (without accessing the free text content of primary care records), and to understand the pathway to child disability diagnosis. We have highlighted the clinical practice of deferred disability diagnosis during the preschool period. For data systems with linked mother and child health records, our strategy could be used to investigate regional variation in time to diagnosis and thus variation in practice. Further our strategy can be used in the investigation of the impact of diagnostic uncertainty on caregiver health. Despite caregiver statements that the period of disability identification and diagnosis are highly stressful, there is little empirical research on this period in relation to caregiver ill-health (Sloper, 1993). Studies have looked at caregiver adjustment but encompassing a wider child age range (Noojin, 1997; Sanders, 1997; Witt, 2003). The longitudinal investigation of changes in caregiver adjustment and health over time, and at key points of disability identification, diagnosis, and transitions between preschool, school and adult services have not been investigated. By identifying key points of caregiver burden and whether these vary by disability diagnosis, services and interventions that support families at high-risk intervals across the life course could be developed.

## Conclusion

We have developed a strategy for identifying preschool aged children with developmental disabilities via primary care records. We have shown that by using a two-part case ascertainment approach which combines strategies that identify probable and potential disability, a realistic estimate of developmental disability in children aged 0-5 can be obtained. However, questions remain about misclassification error and without accessing additional information about the children, disability severity cannot be assessed using the strategy.

## Data availability

Scientists are encouraged and able to use BiB data, which are available through a system of managed open access. The steps below describe how to apply for access to BiB data.

- Before you contact BiB, please make sure you have read our Guidance for Collaborators. Our BiB executive review proposals on a monthly basis and we will endeavor to respond to your request as soon as possible. You can find out about the different datasets which are available here. If you are unsure if we have the data that you need please contact a member of the BiB team ( borninbradford@bthft.nhs.uk).
- Once you have formulated your request please complete the ‘Expression of Interest’ form available here and send to the BiB Programme Director ( rosie.mceachan@bthft.nhs.uk).
- If your request is approved. we will ask you to sign a collaboration agreement and if your request involves biological samples we will ask you to complete a material transfer agreement.
