# Supplementary material for: An approach to identifying young children with developmental disabilities via primary care records
Source: Wellcome Open Res. 2021 Sep 22;6:189. Originally published 2021 Jul 26. [Version 2] doi: 10.12688/wellcomeopenres.17051.2 (PMC8822140; doi:10.12688/wellcomeopenres.17051.2)
Supplement: Peer review history

# Peer review: Reviewer response for version 2

**DOI:** 10.21956/wellcomeopenres.19031.r48081

**Published:** 2022-02-07

**Authors:** Paul H. Lipkin

**Affiliations:** 1 Kennedy Krieger Institute, Johns Hopkins School of Medicine, Baltimore, MD, USA

The authors present a cogent and novel method for utilizing early childhood public health data to determine prevalence of developmental disability, a longstanding known challenge. While these conditions represent large portions of the population with unique medical needs as well as special educational, therapeutic, and financial needs, they lack clear precise definitions or biologic markers, with current definitions differing by location (local, regional, national) as well as remaining imprecise. Better precision and uniformity would offer opportunities for community, region, and national comparisons that might not only allow better planning and implementation of service delivery (healthcare, education, community, etc.) but also benefit research aimed at understanding etiology and identifying effective treatments.

 This manuscripts offers a well laid out means for identifying prevalence of these disabilities in a birth cohort in an exploratory fashion for other public health researchers to consider. In so doing, the results reflect comparisons of this methods derived data to data sets that used other methods, and demonstrate a validation of this method in its similar results, but also offer some benefits to this approach in the discussion. The exploratory nature of this paper is well reflected in both the title and the text. 

 The following critiques do not render this paper any less useful but should be considered by the authors or reader.

**Methods:**

1. The authors report the exclusion of disability conditions when fewer than five children were identified, for protection of privacy. The impact of this decision on the results merits discussion in the Discussion section, including consideration for other researchers using this method on similar or larger cohorts. Would some conditions be un-identified and what would the impact be?
2. Demographics- The racial and ethnic demographics appear unique to the UK and appear to exclude many under-represented and often under-studied minority groups. For a non-UK resident, it is unclear what "White" represents as well as "Pakistani". What about those who immigrated from other continents (Africa, Asia, Latin America)? And are these distinctions necessary for such research or do they contribute to public discrimination?

**Results:**

1. In the first sentence under "Probable disability", it states 83 children were identified (or 85/10,000), yet in Figure 1, the numbers do not seem consistent. Please clarify the totals either in the text, tables, or figures. I assume the 83 = 39 + 44 in the figure but had to re-read the numbers, tables, and figures to be sure, since each used a different denominator (Figure used total cases, I assume, while Table 3 used "per 10,000". Consistency or clear explanation are preferred. I struggled similarly with Figure 2 in comparing the "n"s on the figure with the numbers in the column.
2. On Table 4, as noted in my Methods comment, I have concerns around the ethnicity data generally. It emerged from the row in this table, where the only choices were "White British" and "Pakistani". Are those with roots in Pakistan not British? What is "White"? Are there no people in Bradford from other continents? Are they not all British?

**Discussion:**

1. In the first sentence of the 3rd paragraph ("An additional finding..."), the authors identify a potential flaw in their method and discuss it as warranted. There is a reference to an article that their conclusion is based on. This highlights the weakness of the definition of "global developmental delay" which offers a refuge for imprecision in diagnosis and leads to public health research problems being addressed here. The authors are credited for some discussion to this point and the impact.
2. "...a greater number of mothers of children with ASD had higher education..."- Is it that higher educated mothers are more assertive or persistent or do they have greater access to diagnostic care due to related wealth tied to their education?
3. Strengths and limitations: First paragraph- By definition, developmental disabilities require time (development) to emerge and be identifiable, creating the research problem being examined here. Would the authors consider offering a "best age" for studying prevalence of DD, rooted in their findings?

While these critiques highlight questions for the investigators, they deserve praise and close examination of the strengths and limits of their research. The Conclusion is perfect, as is this sentence under the Strengths and Limitations section ("As these are common challenges in using...") which sums up the value of this paper.

Is the work clearly and accurately presented and does it cite the current literature?

Yes

If applicable, is the statistical analysis and its interpretation appropriate?

I cannot comment. A qualified statistician is required.

Are all the source data underlying the results available to ensure full reproducibility?

Yes

Is the study design appropriate and is the work technically sound?

Yes

Are the conclusions drawn adequately supported by the results?

Yes

Are sufficient details of methods and analysis provided to allow replication by others?

Yes

Reviewer Expertise:

Clinical early identification of developmental disabilities through screening and evaluation (i.e. developmental surveillance and screening in pediatric care)

I confirm that I have read this submission and believe that I have an appropriate level of expertise to confirm that it is of an acceptable scientific standard.

# Peer review: Reviewer response for version 1

**DOI:** 10.21956/wellcomeopenres.18832.r45138

**Published:** 2021-08-20

**Authors:** Sydney Pettygrove

**Affiliations:** 1 University of Arizona, Tucson, AZ, USA

I believe that the goal of this paper is to present a methodology for using primary care records to identify children with developmental disabilities - but the paper does not clearly express why that would be a useful thing to do. I understand that decreasing the age at diagnosis and ensuring that children are receiving support and services is essential to achieving their full potential but this is less than clear from reading the paper. Please address the question, "Why bother?" more clearly.

 In the Introduction (page 3) the sentence, "the US estimate contains a greater range of conditions than the UK estimate, the US estimate includes a greater range of conditions: attention deficit hyperactivity disorder; intellectual disability" is repetitious.

 In the Discussion section, we find the paragraph beginning, "An additional finding of note was that fewer children in the potential disability group than expected had more than one indicator". My response to this paragraph, in general, is, "OK - what about that?" Again, please state very clearly the response to the question "so what?". What difference does it make that the practice is to give a quite general code in the 0 - 4 year olds, then a definitive diagnosis only later?

 I can appreciate that to bring children in for an evaluation is another whole study, but this paper really suffers from its lack of a gold standard.  Without that, it is essentially impossible to evaluate the utility of this method. The fact that the prevalence estimate comes close to other prevalence estimates is nice, but what does that really tell us? Do the children identified through these methods really have developmental disabilities and what is the severity for those who do? I would like to see a follow up study involving bringing in a sample of these children for clinical evaluation to provide a more objective view of the utility of the proposed method of identification. I am not necessarily saying that this sort of validation sub-study is a requirement for publication, but I would like to see this concept included in the discussion section for future directions for research.

 These tables would not get a passing score in my class. Why do you run together different types of information in the same column? This just makes the tables more difficult to decipher. It also means that you have parentheses cluttering up the body of the table making it hard to read.

Is the work clearly and accurately presented and does it cite the current literature?

Partly

If applicable, is the statistical analysis and its interpretation appropriate?

Partly

Are all the source data underlying the results available to ensure full reproducibility?

Yes

Is the study design appropriate and is the work technically sound?

Partly

Are the conclusions drawn adequately supported by the results?

Partly

Are sufficient details of methods and analysis provided to allow replication by others?

Yes

Reviewer Expertise:

Epidemiology of developmental disabilities

I confirm that I have read this submission and believe that I have an appropriate level of expertise to confirm that it is of an acceptable scientific standard, however I have significant reservations, as outlined above.

# Response

**Published:** 2021-09-13

**Authors:** Sarah Masefield

**Affiliations:** University of York, UK, UK

Thank you, Prof Pettygrove, for your review of our manuscript. We have made the recommended revisions and think the manuscript is greatly improved. A summary of the changes we have made in response to each of your comments is provided below.

 We acknowledge that the important ‘why bother’ message was missing from the abstract and the importance of early diagnosis for families could be strengthened in the introduction. In both places, we have added statements stating the potential impact of delayed diagnosis on families’ access to appropriate support and resources. We believe that it is now clear from the introduction that it is valuable to researchers, health and social care commissioners and families that accurate estimates of disability prevalence in young children can be obtained from the analysis of primary care records, and of the benefit of early diagnosis of disability to families.

 We have revised the sentence on page 3 identified as repetitious, identifying only the additional conditions included in the US estimate.

 In responding to your ‘so what?’ question of why it matters that fewer children in the potential disability group than expected had more than one indicator of disability, we have suggested possible explanations for the observed finding as we believe it could indicate issues in the diagnostic process.

 Thank you for identifying and suggesting the need for a clear statement of research needed to further explore and strengthen our approach and findings. Although it was not possible within the scope of our study to use a gold standard assessment, we acknowledge this as a significant limitation and elaborate on this in our strengths and limitations section. Hopefully we may be able to perform this study ourselves in future.

 Thank you for prompting us to improve the accessibility of our figures. We have revised the presentation of figures 2-5 – they are now side by side bar charts with the labels below and not on the bars).

# Peer review: Reviewer response for version 1

**DOI:** 10.21956/wellcomeopenres.18832.r45139

**Published:** 2021-08-02

**Authors:** Karen Horridge

**Affiliations:** 1 South Tyneside and Sunderland NHS Foundation Trust, Sunderland, UK

Thank you for the opportunity to review this important manuscript. The authors use routinely available primary care coded data to quantify confirmed and suspected developmental disabilities in children aged 0-5 years.

 The authors use Read codes to identify diagnoses and possible diagnoses. These are being phased out in favour of SNOMED-CT codes, so any researchers wishing to replicate the work in future would need to be able to map the Read coded terms to those available in the SNOMED-CT browser.

 One of the challenges in this area of practice is the lack of harmonisation of terminologies used by clinicians and this is likely to vary between clinicians and localities. Thus, whilst in Bradford the term 'developmental delay' is used a lot, other clinicians and services avoid this, rather favouring Early Developmental Impairment or Provisional Intellectual Developmental Disorder (ICD-11). It would be unusual for a child aged four years or under to have a confirmed learning disability, as assessments at this age are unreliable due to developmental changes.

 These differences in terminologies would need to be taken into consideration if others were wanting to replicate the study in different settings, but mapping should be possible, as long as definitions of the terms used are clear.

 it is surprising that 'possible autism spectrum' was not part of the provisional language used, but again, it may be that the local clinical service uses different terms for children undergoing autism assessments in the early years.

 In all, the authors are to be commended to tackling an area where there is a paucity of robust, population research and hopefully others will build on the knowledge gained.

Is the work clearly and accurately presented and does it cite the current literature?

Yes

If applicable, is the statistical analysis and its interpretation appropriate?

Yes

Are all the source data underlying the results available to ensure full reproducibility?

Yes

Is the study design appropriate and is the work technically sound?

Yes

Are the conclusions drawn adequately supported by the results?

Yes

Are sufficient details of methods and analysis provided to allow replication by others?

Yes

Reviewer Expertise:

Paediatric disability Population data Complexity of needs Learning disability

I confirm that I have read this submission and believe that I have an appropriate level of expertise to confirm that it is of an acceptable scientific standard.

# Response

**Published:** 2021-08-02

**Authors:** Sarah Masefield

**Affiliations:** University of York, UK, UK

Thank you Dr Horridge for your positive review of our manuscript. Thank you also for raising some helpful cautions around the variability of terminology between settings which will be of benefit to readers in contexts where different terms and practices may be the norm, including where different clinical coding languages are used.
